# Effects of salt stress levels on nutritional quality and microorganisms of alfalfa-influenced soil

**DOI:** 10.7717/peerj.11729

**Published:** 2021-07-14

**Authors:** Qiang Lu, GenTu Ge, DuoWen Sa, ZhiJun Wang, MeiLing Hou, Yu Shan Jia

**Affiliations:** 1 College of Grassland and Resources and Environment, Inner Mongolia Agricultural University, Hohhot, Inner Mongolia Autonomous Region, China; 2 College of Agriculture, Inner Mongolia University for Nationalities, Tongliao, Inner Mongolia Autonomous Region, China

**Keywords:** Alfalfa, Salt stress, Protein, Microorganism

## Abstract

**Background:**

Globally, there is a large amount of salinized land. These soils have varying degrees of salt stress, causing ionic toxicity and osmotic stress on plants. However, it is not clear how different degrees of salt stress affect plant nutrients and microbial communities. Thus, a comprehensive understanding of plant major nutrients and microbial communities response to salt stress is desirable.

**Results:**

We analyzed the main nutrients of the salt-tolerant ZhongMu No. 3 alfalfa variety planted in a salt stress environment. In mild and moderate group, the protein content and fatty acid content of alfalfa were the highest, indicating the best nutritional value. The severe group of salt stress affected the growth and development of alfalfa, as manifested by a decrease in the nutritional quality of alfalfa. *Pseudomonas* and *Sphingobacterium* that from alfalfa stem and leaf endophytes also increased with an increase in salt stress. In contrast, *Sphingomonas*, *Methylobacterium*, and *Rhizobium* decrease with increasing salt stress. *Methylobacterium* and *Rhizobium* have extremely significant differences in response to salt stress, and *Exiquobacterium* also shows significant differences.

**Conclusions:**

Soil salinity would be an important factor beyond which alfalfa nutrient quality and microbial community structure change. This study identified key levels of salt stress that may affect the nutrient quality and microbial community structure. These findings enhance our understanding of the effects of salt stress on the nutritional quality of alfalfa and provide a reference for the sustainable use of salinized soil in the future.

## Introduction

Soil salinity stress is a serious abiotic factor, which restricts the growth and accumulation of nutritional quality in crops (Munns & Tester, 2008). According to statistics from UNESCO and FAO, saline-alkali soil resources are distributed in more than 100 countries around the world, and there is a global saline-alkali land area of 955 million hm^2^ (Wang et al., 2017). Producing more plant resources with limited and salt-affected land has become increasingly important (Flowers & Colmer, 2017). Thus, understanding the nutritional quality of plants and the effects of salt stress on microorganisms is a priority for addressing the growing supply of global forage resources (Witzel et al., 2009).

Salt stress and ionic effected in the soil result in low plant absorptive capacity, nutrient deficiencies, and plant ion imbalance (Shi et al., 2008). Salinity affected the growth and development of plants through osmotic stress and the deleterious effects of high Na^+^ and Cl^−^ (Farissi et al., 2011). The chloroplast is one of the most sensitive organelles under salt stress. Salt stress damaged the chloroplast structure, reduced the chlorophyll content, and caused the photosynthetic capacity of the plants to weaken. As a result, crop yields and nutrients quality were reduced. Pushpam & Rangasamy’s, (2000) research on rice, chloroplast content showed an upward trend when it was at a lower salt stress level. . Moreover, from the perspective of plant nutrition metabolism, with the increase of salt stress, the content of proline in crops also gradually increased (Sanada et al., 1995). In the study by Lin et al. (2018), Tomato’s soluble sugar and soluble protein eventually got an increasing trend in response to salt stress.

In particular, competition for some ionic factors such as K^+^, Na^+^, and Cl^−^ would led to plant nutrient imbalance (El-Sharkawy et al., 2017). Besides, it has been documented that excessive concentrations of Na^+^ in plant tissues prevent nutrient and osmotic balance, leading to specific ionic toxicity (Hariadi et al., 2010). Therefore, salt stress was a major hazard to plants and prevents the effective utilization of saline areas (Rodr et al., 2005). Many plants, such as alfalfa (*Medicago sativa* L.), which has high levels of crude protein, digestible nutrients, and minerals, had been used to ameliorate this problem (Jia et al., 2017). Alfalfa can be planted on saline soils. As a high-quality pasture resource, it could adapt well to the environment in saline-alkali lands (Diaz et al., 2018). Alfalfa cultivation enabled the production of high-protein forage feed (Stritzler et al., 2018) to support the development of animal husbandry (Suyama et al., 2007).

Endophytes microorganisms in plants were important for plant growth, and their secretions may have significant benefits for plants, especially in saline-alkali areas (Jayne, 2012). Pandey et al. (2012) inoculated *Pseudomonas aeruginosa* PW09 in the wheat endophytic bacterium. With salt stress, application of the *P. aeruginosa* PW09 strain induced accumulation of free proline and increased activity of defenserelated enzymes like polyphenol oxidase. After soybeans were inoculated with *P. simiae*, the content of soluble sugar and proline increased in response to stress (Vaishnav & Choudhary, 2018). *Yarrowia lipolytica* was reported that its been inoculated on corn. The salt tolerance of corn has been greatly improved. In addition, the proline content of corn has also increased (Farzana et al., 2019). The Hetao Plain was a region with a large area of saline soil in China (Yang et al., 2016). In this unique environment, salt-tolerant and halophilic bacteria grow and the resulting microbial population is likely to contain abundant new species resources. Take the perspective of sustainable high-quality agricultural production, how to use these saline-alkali lands has attracted great attention from the world. Therefore, it is very urgent to understand the effect of salt stress on the nutritional quality of forage and clarify the response mechanism of crops to salt stress. Our study aimed to identify the effects of salt stress on alfalfa and to further understand the role of the endophytes microbial community in alfalfa growth.

## Materials & Methods

### Experimental site

This study was conducted in a field site in Baotou City, Inner Mongolia Autonomous Region, China (coordinates 40 ^∘^17′N, 111 ^∘^27′E). The experimental site was located on the north bank of the Yellow River, which is in the Hetao Plain. The study site was a typical saline area.

### Alfalfa Preparation

ZhongMu No.3 alfalfa was planted on four kinds of land in the experimental base, and it was divided into CK, mild, moderate, and severe according to their salinity. The physical and chemical properties of the soil are shown in Table 1. ZhongMu No.3 alfalfa was a variety selected by the Beijing Institute of Animal Science and Veterinary Medicine of the Chinese Academy of Agricultural Sciences. Its characteristics are strong salt resistance, good palatability, high digestibility, nutrition, and rich in value (Ma et al., 2017). These seeds were also provided by the Beijing Institute of Animal Science and Veterinary Medicine of the Chinese Academy of Agricultural Sciences.

**Table 1:** The conditions of different salinized soils.

Indictors	Na^+^(g/kg)	K^+^(g/kg)	Cl^−^(g/kg)	SO_4_^2−^(g/kg)	pH	EC (mS/cm)
CK	0.11 ± 0.0058c	0.027 ± 0.0005d	0.051 ± 0.001c	0.008 ± 0.02b	7.4 ± 0.21b	0.21 ± 0.024a
Mild	0.15 ± 0.0035b	0.031 ± 0.0011c	0.119 ± 0.004b	0.023 ± 0.013a	8.4 ± 0.08a	0.59 ± 0.10b
Moderate	0.16 ± 0.0020b	0.035 ± 0.001b	0.125 ± 0.0023b	0.024 ± 0.0049a	8.6 ± 0.11a	1.35 ± 0.01c
Severe	0.25 ± 0.019a	0.041 ± 0.001a	0.203 ± 0.0034a	0.027 ± 0.015a	8.7 ± 0.34a	2.3 ± 0.29d

**Notes.**

Numbers in a column followed by different lowercase letters differ at *P* < 0.05.

EC electrical conductivity

The alfalfa used in the experiment was cutting at the initial flowering stage, and the height of the stubble for cutting was five cm. The collected alfalfa were surface-disinfected with 70% ethanol, 2% sodium hypochlorite, followed by five times rinsing with sterile distilled water for 2 min. Alfalfa of 10 g that included leaves and stems sample was taken from each foil in each treatment group and immediately chopped into 1—2 cm lengths (without root). Then use a sterile grinder to ground into a powdery shape to better release endophytes. 500 g samples from each treatment group were put into kraft paper bags and dried for testing nutritional contents. There were three replicates for each treatment.

### Analysis of soil characteristics and alfalfa chemical composition

Soil samples were air-dried indoors, soil EC (electrical conductivity) was determined by EC Meter (FieldScout EC 110 Meter), Na^+^ and K^+^cations were determined by atomic absorption spectrometry (AA-6800, Daojin, Japan) (Li et al., 2019), SO${}_{4}^{2- }$ and Cl^−^ were determined by ion chromatography (IC-2000; Dionex, Sunnyvale, CA, USA) (Li et al., 2019). The soil pH was determined with a PHS-3C type acidity meter.

The dry matter (DM) content of fresh plant samples was oven-dried (ULM 800; GmbH, Schwa Bach, Germany) at 65 ^∘^C for 48 h. Dried samples were ground to one mm particles and crude protein (CP), soluble protein (SP), neutral detergent fiber (NDF), acid detergent fiber (ADF), lignin, and fatty acids (FA) were analyzed by near-infrared reflectance spectroscopy (NIRS). All results are reported as % dry mass (% DM). The spectra were analyzed using a large dataset of calibration samples from different kinds of grasslands available from the Institute VDLUFA Qualitätssicherung NIRS GmbH, Kassel, Germany.

### High throughput sequencing of microbial population

Microbial DNA was extracted from fresh alfalfa endophytes microorganisms through the following steps. 10 g of plant samples that has been ground were mixed with 90 mL of sterile water and then treated with a table concentrator at 120r/m for 2 h. The sample was then filtered with carbasus and the liquid was centrifuged at 10,000 rpm for 10 min at 4 ^∘^C. The supernatant was abandoned and the pellet was suspended in one mL of sterile water solution. The precipitate was used for DNA extraction.

Microbial DNA of alfalfa samples was extracted using the E.Z.N.A.®soil DNA Kit (Omega Bio-Tek, Norcross, GA, USA) according to the manufacturer’s protocols. The final DNA concentration and purification were determined by NanoDrop 2000 UV-vis spectrophotometer (Thermo Scientific, Wilmington, DE, USA), and DNA quality was checked by 1% agarose gel electrophoresis. The V3-V4 hypervariable regions of the bacteria 16S rRNA gene were amplified with primers 338F (5′-ACTCCTACGGGAGGCAGCAG-3′) and 806R (5′-GGACTACHVGGGTWTCTAAT-3′) by thermocycler PCR system (GeneAmp 9700, ABI, USA). The PCR reactions were conducted using the following program: 3 min of denaturation at 95 ^∘^C, 27 cycles of 30 s at 95 ^∘^C, 30s for annealing at 55 ^∘^C, and 45s for elongation at 72 ^∘^C, and a final extension at 72 ^∘^C for 10 min. PCR reactions were performed in triplicate 20 μL mixture containing 4 μL of 5 × FastPfu Buffer, 2 μL of 2.5 mM dNTPs, 0.8 μL of each primer (5 μM), 0.4 μL of FastPfu Polymerase and 10 ng of template DNA. The resulted PCR products were extracted from a 2% agarose gel and further purified using the AxyPrep DNA Gel Extraction Kit (Axygen Biosciences, Union City, CA, USA) and quantified using QuantiFluo^*TM*^-ST (Promega, USA) according to the manufacturer’s protocol.

Raw fastq files were demultiplexed, quality-filtered by Trimmomatic, and merged by FLASH with the following criteria: (a) the reads were truncated at any site receiving an average quality score <20 over a 50 bp sliding window; (b) primers were exactly matched allowing 2 nucleotide mismatching, and reads containing ambiguous bases were removed; (c) sequences whose overlap was longer than 10 bp were merged according to their overlap.

Operational taxonomic units (OTUs) were clustered with 97% similarity cutoff using UPARSE, and chimeric sequences were identified and removed using UCHIME. The taxonomy of each 16S rRNA gene sequence was analyzed by RDP Classifier algorithm against the Silva (SSU123) 16S rRNA database using a confidence threshold of 70%. We uploaded the sequences data in the NCBI under the accession number PRJNA560790.

### Statistical Analysis

SAS 9.3 software was used to analyze differences between treatment means using Tukey’s multiple comparison test, with a significance level of *P* < 0.05. The data from high throughput sequencing and Non-metric multidimensional scaling (NMDS) were analyzed on the Majorbio I-Sanger Cloud Platform (http://www.i-sanger.com).

## Results

### Soil conditions of the study site

Data on field salinity is shown in Table 1. Soil pH, EC, Na^+^, and Cl^−^ were affected by soil salinization. The soil properties of the CK area were significantly different from other test areas (*P* < 0.05). The salt stress of three groups were higher than CK, and the differences between the mild area and the moderate area were small. The soil EC in the mild area was 0.59 mS/cm, whereas the EC in the severe area was 2.3 mS/cm. The content of Na^+^ in CK was 0.11 g/kg, which was significantly different from other treatment groups. The content of Na + in the severe group was more than twice that of the CK group, which was 0.25 g/kg. Overall, the cations and anions of the CK group were significantly different from those of the other groups, while the contents of Na^+^, Cl^−^, SO_4_^2−^, pH of mild and moderate were not significant.

### The chemical composition of alfalfa in fields under salt stress

**Figure 1:**
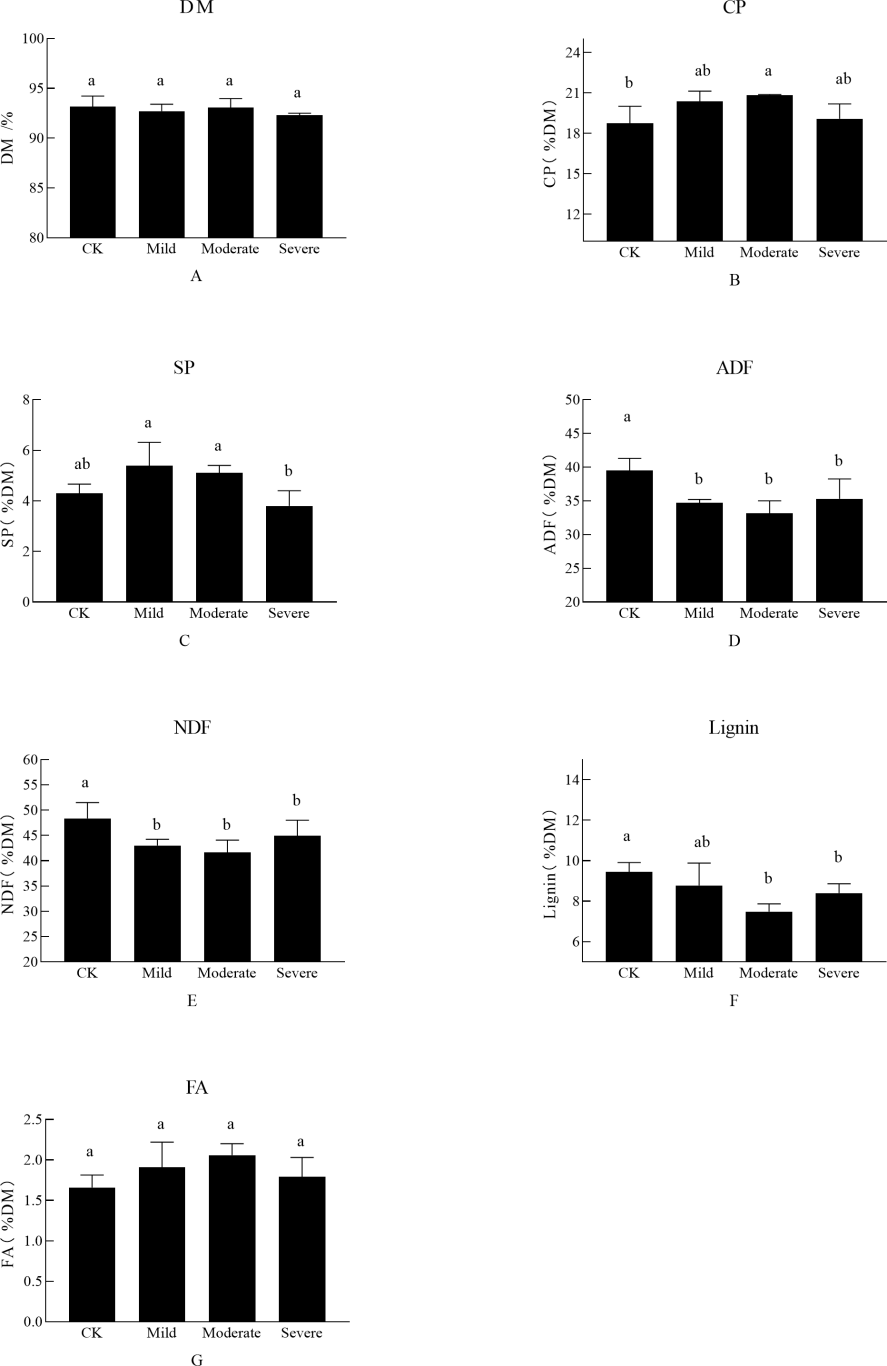
The effects of salt stress on nutritional quality of alfalfa (A–G). (A) Dry matter (DM), (B) crude protein (CP), (C) soluble protein (SP), (D) acid detergent fiber(ADF), (E) neutral detergent fiber (NDF), (F) Lignin, (G) fatty acid (FA). Bars indicate standard error of the means.Different lowercase letters differ at *P* < 0.05.

As shown in Fig. 1, the abiotic stress of salt on alfalfa nutrition was obvious. There was no significant difference in the DM of each treatment, CP which is one of the most important indicators for judging the quality of alfalfa was significantly different between treatments. There was a significant difference between the three treatment groups and the CK, which was the salt stress alfalfa CP was higher than CK. The CP was up to 20.85% DM in moderate stress. At this time, the SP content under moderate salt stress treatment was 5.1% DM, which was also at a high level. However, when the soil EC reached severe salt stress (i.e., Na^+^ was 0.25 g/kg and Cl^−^ was 0.203 g/kg), the CP decreased, and SP content also decreased. FA did not show significant differences when faced with different salt stresses. There was no significant difference between NDF and ADF in salt stress (*P* > 0.01). When alfalfa was planted in moderate and severe site, it was difficult for the alfalfa to maintain it’s ion balance, resulting in a decrease in SP content, which in turn affected CP content. The severe group of salt stress affected the growth and development of alfalfa, as manifested by a decrease in the nutritional quality of alfalfa.

### Microbial community composition under different salt stress

**Figure 2:**
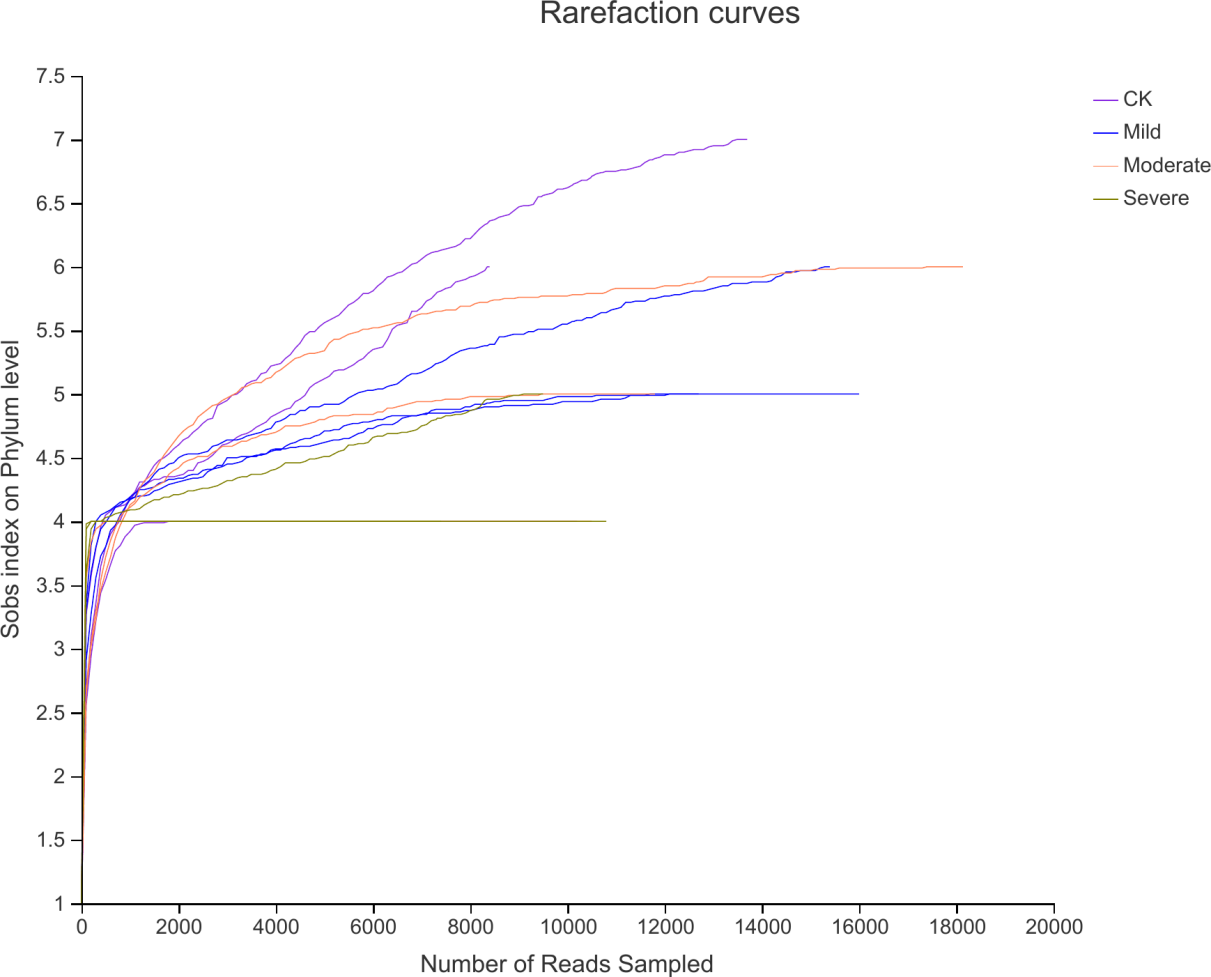
Rarefaction curves for alfalfa samples under salt stress. CK, control; Mild, Moderate, Severe, treated under salt stress.

High-throughput assays were performed for variable regions 3 and 4 of 16srDNA, the rarefaction curve (Fig. 2) showed that the sequencing work was relatively comprehensive in covering the bacterial diversity, as the rarefaction curves tended to approach saturation. To identify the bacterial diversity of alfalfa treated with different salt stresses (Table 2), The coverage of all samples were greater than 99%, indicating that the sequencing breadth was relatively comprehensive, and the data was sufficient to represent the characteristics of bacterial microbial communities. According to the number of OTU and CHAO index, the abundance of endophytes bacterial communities under different salt stress treatments were different. The OTU number of CK was 154, this was less than mild group. But more than the moderate and severe groups. Similarly, the Shannon index was also the highest for CK. This also showed that the biological environment of the CK group was more diverse and more abundant.

**Table 2:** Richness and diversity indices of microbial communities of alfalfa. Operational taxonomic units (OTU). The Shannon index illustrates the heterogeneity of microbial communities; the Ace index is used to estimate the OTU number of in a community, indicating the richness of the microbial community; the Chao index is used to assess the total number and richness of species in a community.

Samples	OTU number	Shannon	Ace	Chao	Coverage
CK	154	2.8	146.66	139.79	0.99
Mild	173	2.35	161.89	158.27	0.99
Moderate	147	2.1	138.61	159.24	0.99
Severe	130	2.54	119.03	113.36	0.99

**Notes.**

OTU Operational taxonomic units

**Figure 3:**
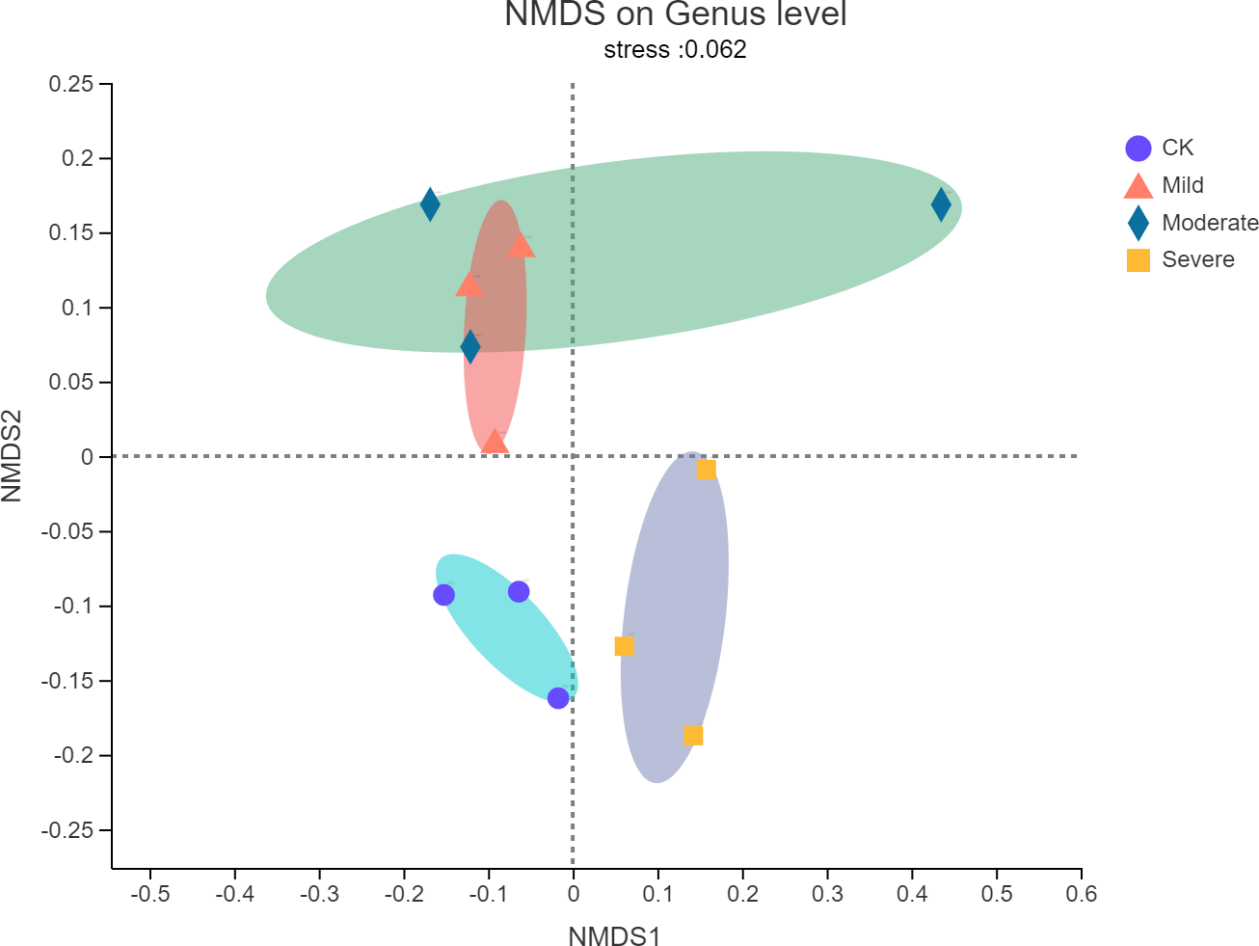
Non-metric multidimensional scaling (NMDS) of bacterial communities of alfalfa plants based on different salt stresses. The ellipse represents the differences between microbial communities in salt stress treatment.

Non-metric multidimensional scaling (NMDS) analysis was used to visualize the differences in the distribution and structure of bacterial microbial communities after different salt stress treatments. As shown in Fig. 3, there were significant differences in the endophytes microorganisms of alfalfa after treatments. The NMDS resulted have a stress value of 0.062, which meant that the NMDS analysis results indicate that there was no stress in 2-dimensional representation. Mild salt stress and moderate salt stress had similar microbial community structure. In the severe group, the microbial community structure changes from a structure with a higher similarity. There was no overlap with the original community.

**Figure 4:**
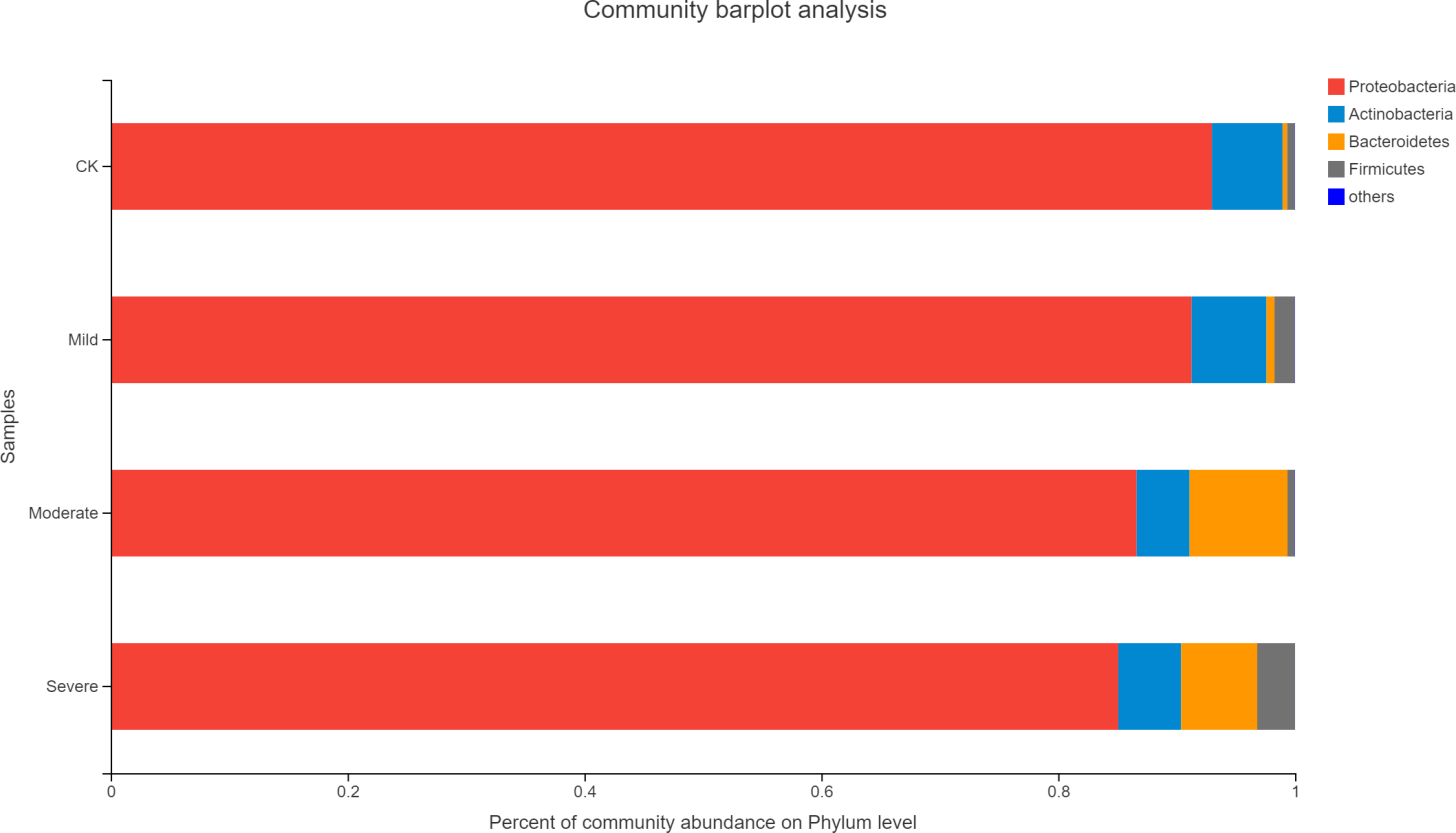
Relative abundance of bacterial at the phylum level. CK, control; Mild, Moderate, Severe, treated under salt stress. There are three triplicates for each treatment.

**Figure 5:**
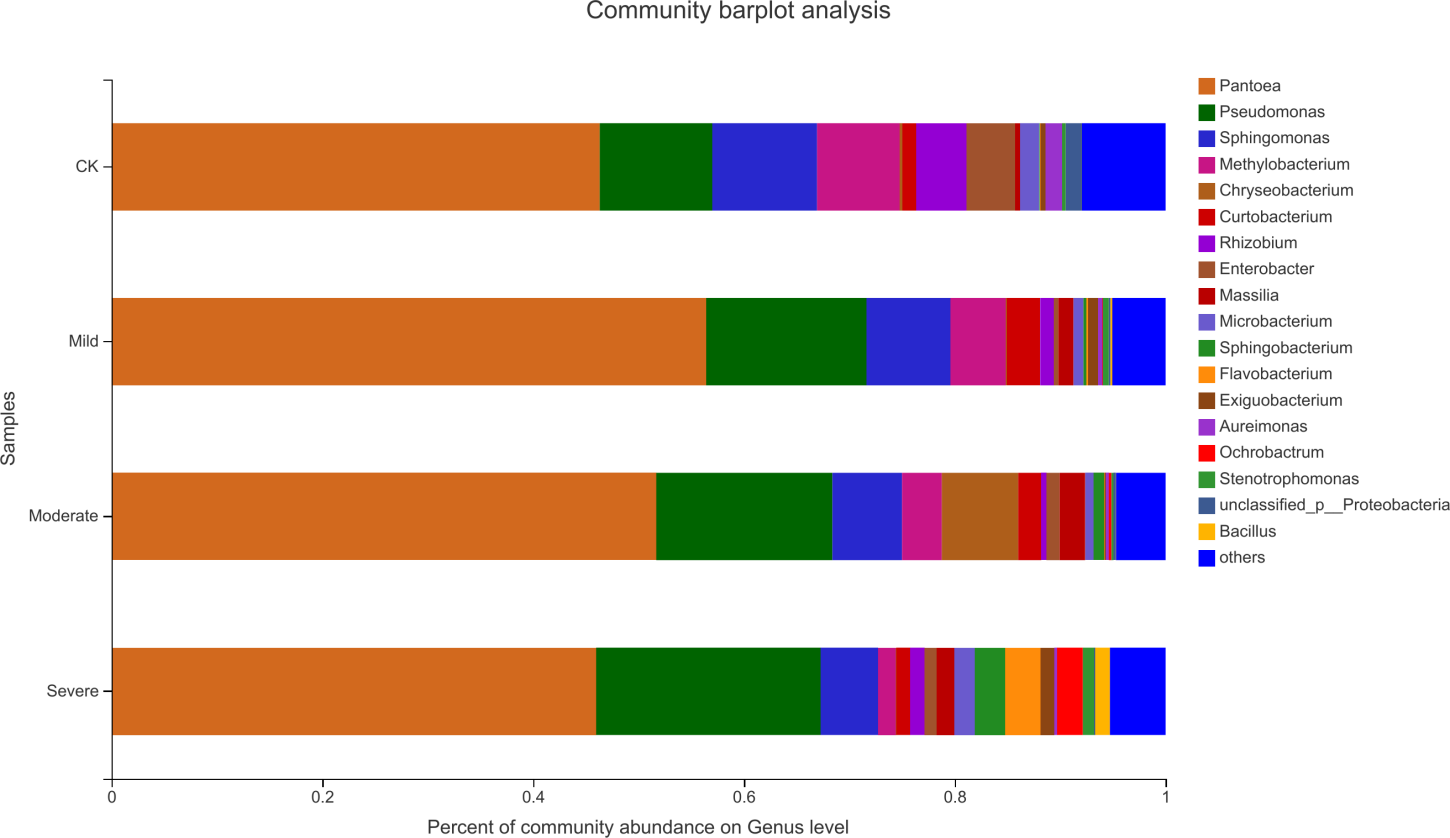
Relative abundance of bacterial at the genus level. CK, control; Mild, Moderate, Severe, treated under salt stress. There are three triplicates for each treatment.

Alfalfa microbial community composition is shown in Figs. 4 and 5, showing the distribution of at the genus level under different salt stress levels. *Proteobacteria* was the most abundant bacterial population. CK (92.9%) had 7.83% more *Proteobacteria* than the severe salt stress treatment (85.07%). As salt stress increases, the proportion of *Proteobacteria* decreased monotonically. Among the four groups of salt stress treatments, *Pantoea* had the highest abundance, followed by *Pseudomonas*. With the increasing of salt stress, the abundance of *Methylobacterium* and Sphingo*monas* decreased gradually. There was a big significant differences between CK and severe in Paenibacillaceae, Aurantimonadaceae, Caulobacteraceae, Nocardia, Kineosporiaceae, Comamonadaceae, Sphingobacteriaceae, Brucellaceae and Bacillaceae. Among them, the abundance of Orangeaceae, Petiaceae, Nocardaceae, Kineosporiaceae and Comamonadaceae were decreased compared with CK, and the others were increased. *Planococcus* can develop perchlorate-specific stress adaptations that were not (or only to a lower extent) used to counteract high Na^+^ concentrations. The response of plant microorganisms to different salt stresses was very interesting and beyond expectation.

**Figure 6:**
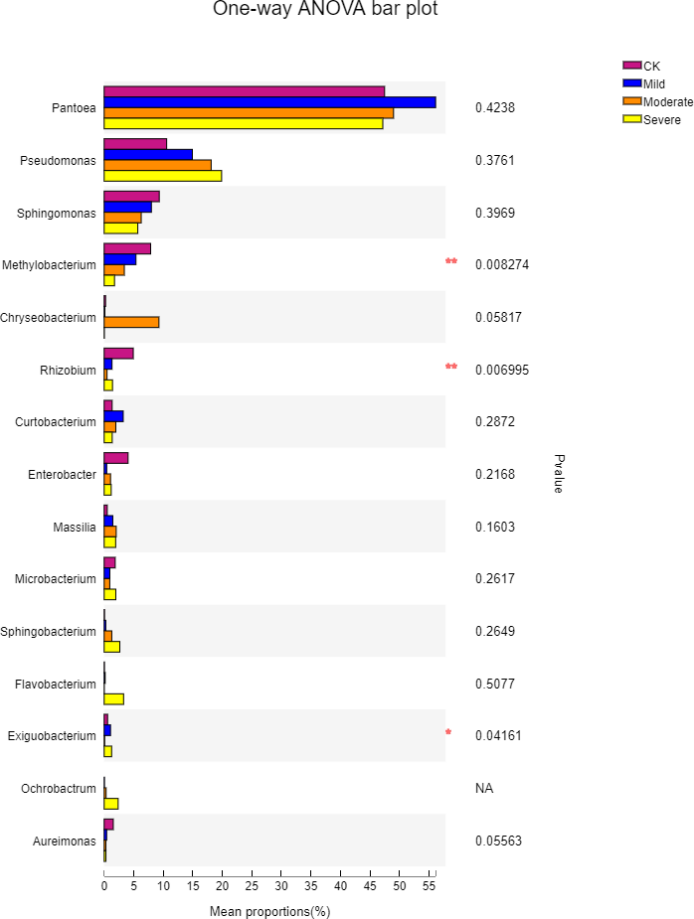
One-way ANOVA bar plot difference significance test between four groups at the genes. CK, control; Mild, Moderate, Severe, treated under salt stress. There are three triplicates for each treatment. An asterisk (*) is representative of *p* < 0.05, with significance; two ampersands (**) is representative of *p* < 0.01, with extreme significance.

To further understand the effects of different salt stress levels on the microbial genus, we performed a one-way analysis of variance for the top 15 genera in the abundance of different treatments. The results are shown in Fig. 6. With the gradual increase in salt stress, *Pseudomonas*, *Sphingomonas*, *Methylobacterium*, *Sphingobacterium*, and *Aureimonas* showed strong response patterns. *Pseudomonas* and *Sphingobacterium* increased with salt stress increased, which may be due to the fact that these two genera prefer to survive and reproduce in saline environments. In contrast, *Sphingomonas*, *Methylobacterium,* and *Aureimonas* decreased with increasing salt stress and showed salt-tolerant properties. It can be seen from Fig. 6 that although the microbes in the first 15 genus have a large difference in their responses to different salt stress levels, there was one genus with a significant difference, which was *Exiguobacterium*. The genus with extremely significant differences were *Methylobacterium* and *Rhizobium*. *Methylobacterium* had a content of 1.7% under severe salt stress. This indicated that *Methylobacterium* has better salt tolerance and also helps alfalfa to better cope with salt stress. *Exiguobacterium* was widely used for the industrial production of enzymes. In the CK, the content of *Exiguobacterium* was less at 0.57%.

**Figure 7:**
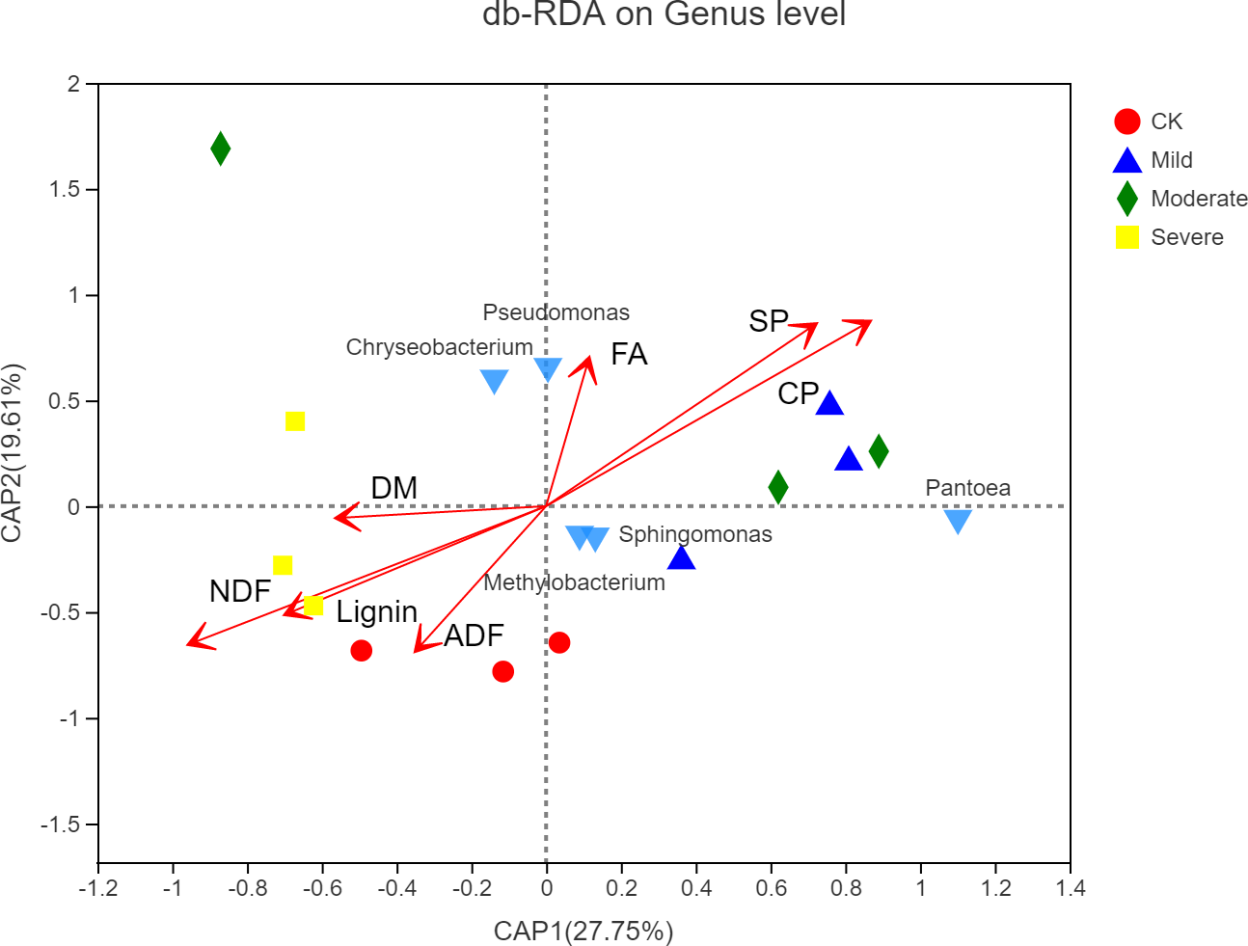
Distance-based redundancy analysis of microbial community and alfalfa chemical composition. CK, control; Mild, Moderate, Severe, treated under salt stress. There are three triplicates for each treatment.

As shown in Fig. 7, the microbial factor analysis (db-RDA) diagram shows the correlation between microbes, samples and major nutritional indicators at the genus level. Samples under the same salt stress were close, while samples under different salt stress were far. The microbial community structure of moderate and mild group was similar, and the microbial community structure of CK was different from that of the mild and the moderate, indicating that salt stress would change the structure of microbial community. The CP, SP and FA were positively correlated, and they were negatively correlated with lignin, ADF and NDF, respectively. The CP, SP, FA vector points to mild and moderate, indicating that CP, SP, FA were positively correlated with the mild and the moderate. The correlation between *Sphingomycetes, Methylobacterium* and CP, SP, and FA was high, while the correlation between *Pseudomonas* and *Chryseobacterium* and FA was high.

## Discussion

### Alfalfa nutrition in fields under salt stress

Salt stress affects the normal growth and development of plants and affects the nutrients of plants. Differences between SP in different treatments can be considered as a detoxification effect of plants (Zhu, 2001) and there were also significant differences in SP between mild and moderate salt stress. SP was an important factor in relieving osmotic pressure in response to salt stress (Kanwal, Razzaq & Maqbool, 2019). The SP content in a low salt treatment increased significantly (Oprica et al., 2016), while the SP content in a high salinity growth environment was decreased (Agastian, Kingsley & Vivekanandan, 2000). FA was also important detoxifications for plants to respond to abiotic stresses (Constantino et al., 2013). But it has no significant differences among those treatments, or it may be that the FA represents the total fatty acid, not the finer molecular compound such as exogenous FA, which was protecting the tonoplast of the plants (Zhao & Qin, 2005).

### Bacterial diversity under different salt stress

With an increasing in salt stress, the richness of bacterial communities decreased. The bacterial community shannon index in the moderate salinity treatment group was 2.1, lower than the other groups. In the mild and moderate salt stress group, richness was 158.27 and 159.24, respectively. This may be due to salt stress increasing the taxonomic groups in the bacterial family (Meng et al., 2019a; Wang et al., 2019b). This was very interesting, we need more research to confirm that.

When soil EC of 1.35 mS/cm, the structure of the flora changed greatly. This conclusion was consistent with the findngs of, (Wang et al., 2019b) who reported that the response of bacteria to salt stress was very active and sensitive. This may indicate that soil EC of 1.35 mS/cm was an inflection point, beyond which soil salt stress has a serious influence on plant microorganisms, and there was significant difference in the nutritional quality of alfalfa. This implied that in the face of such sensitive bacterial community structure, there were also different microbial community structures under different salt stresses. This phenomenon can also be compared to habitats in ecology. That was to say, there were many microbial resources and information that we do not understand under salt habitat (Shao et al., 2018). In the face of salt stress, *Proteobacteria* is still the main component of bacteria (Lucas et al., 2013). In addition, the same pattern was seen with *Bacteroidetes* in phylum, which dominates the microorganisms under salt stress (Vasim et al., 2018). In our study, when soil EC reached 1.35 mS/cm, the abundance of *Bacteroidetes* suddenly increased from less than 1% to 8.2%. However, when the soil EC was 0.59 mS/cm, it’s abundance dropped to 6.4%. It was very likely that *Bacteroidetes* were more suitable for living in environments with a certain level of salt content (Yadav & Saxeny, 2018), as was the case with halophilic and salt-tolerant bacteria, but when the soil EC reaches 1.35 mS/cm, *Bacteroidetes* content dropped by 21.9%. When the EC of the soil was too high, the tolerance of some bacteria is reached and inhibits the growth and reproduction of microorganisms.

These halophilic microorganisms significantly changed under salt stress, were likely to play a crucial role in plant growth and development (Yadav & Saxeny, 2018). This may also explain the reasons for the above-mentioned partial alfalfa protein enhancement. Therefore, in the face of abiotic salt stress, in addition to the inherent mechanism of the plant itself in response to salt stress and ionic stress, the endophytes microorganism with the plant (e.g., *Bacteroidetes*) may also help the plant to grow better.

Moreover, the presence of *Rhizobium* and *Pseudomonas* can also alleviate the effects of salt stress on plants (Dilfuza, Dilfuza & Stephan, 2013). However, it was also impossible to rule out the propagation of microorganisms from the roots to the plants. It can also be seen from Fig. 5. that *Rhizobium* was the most suitable for growth in the environment without salt stress, and it’s abundance was also the highest. This may be due to the fact that salt stress has an inhibitory effect on *Rhizobium* (Ahmad et al., 2013), which may also explain the decrease in *Rhizobium* as the increasing salt stress. *Rhizobium* symbiosis had a positive effect on alfalfa salt tolerance by improving the activity of antioxidant enzymes and osmotic adjustment capacity (Wang et al., 2016), at the same time *Rhizobium* symbiosis has positive effects on the salt tolerance of alfalfa by improving antioxidant enzyme activity and osmotic adjustment (Wang et al., 2016). The increase in enzyme activity and the increase in other active ingredients such as catalase (CAT) and peroxidase (POD) may also be factors in the increase in nutrients. Furthermore, the importance of *Rhizobium* to plants was self-evident. However, with the increase of salt stress, the differences between treatments were also significant. For *Rhizobium*, the most suitable part for growth was the root of the sputum. *Methylobacterium* was a genus of *Proteobacteria* that exhibits good colonization properties in a salt-stressed environment (Lee et al., 2015). Also, it contained some substances that can promote plants, such as IAA, cytokinin and so on (Madhaiyan et al., 2006).

In the severe salt stress treatment, the relative content of *Exiguobacterium* increased to 1.26%. *Exiguobacterium*, as a halophile, can grow in a high-salt environment and can produce protease, lipase, amylase, cellulase, mannanase, chitinase The presence of these enzymes can also help plants better adapt to halophytic environments (Johnson et al., 2017). It significantly increased the secondary metabolites in plants by inoculation of *Exiguobacterium* oxidotolerans on brahmi in a saline environment, and helped plants to effectively alleviate the ionic and osmotic stress caused by salt stress Bharti et al., 2013). *Exiguobacterium* has also been detected on corn under salt stress as a salt-tolerant bacteria. Its presence effectively helps alleviate the stress of corn in a salt stress environment (Aslam & Ali, (2018)). In this study, three strains of *Methylobacterium*, *Rhizobium,* and *Exiguobacterium* with significant differences were able to help the abiotic stress in the salt stress environment to a certain extent. The specific mechanism requires further research.

## Conclusions

In this study, an analysis of alfalfa nutrients and microorganisms was carried out under different salt stress levels. We have found that when appropriate salt stress is applied to plants, their nutritional quality is significantly improved. When a limit of soil salinity is exceeded, and alfalfa nutrient quality and microbial community structure change when salinity exceeds this threshold. This is critical for the sustainable use of global saline-alkali resources. At this stage, the nutritional quality of alfalfa is the best and the protein content is the highest. *Methylobacterium*, *Rhizobium,* and *Exiguobacterium* have significant differences under different salt stresses.

##  Supplemental Information

10.7717/peerj.11729/supp-1 Supplemental Information 1 The conditions of different salinized soils

10.7717/peerj.11729/supp-2 Supplemental Information 2 The effects of salt stress on nutritional quality of alfalfa

10.7717/peerj.11729/supp-3 Supplemental Information 3 Raw data for microbe

## Additional Information and Declarations

### Data Availability

The following information was supplied regarding data availability: The raw soil data, nutrient raw data for plant nutrition, and raw data for microbes are available in the Supplemental Files.
